# 

**Published:** 1897-01

**Authors:** 


### CONTENTS.

ORIGINAL COMMUNICATIONS—

A PATHOLOGICAL STUDY OF TYPHOID FEVER. By Charles Minor Blackford, Jr., M.D., Atlanta, Ga. .... 721

REMOVAL OF SIX INCHES OF SMALL INTESTINE. UNION OF THE DIVIDED ENDS BY MEANS OF THE MURPHY BUTTON. By Hunter P. Cooper, M.D., Atlanta, Ga. .... 729

SYPHILIS AS A CAUSE OF ABORTION. By Dr. J. A. Ouimet, Montreal, Can. .... 733

THE VALUE OF CERTAIN THERAPEUTICS IN FUNCTIONAL AND ORGANIC NERVOUS DISEASES. By Curran Pope, M.D., Louisville, Ky. .... 741

SOCIETY REPORTS—

SOUTHERN SURGICAL AND GYNECOLOGICAL ASSOCIATION .... 745

CORRESPONDENCE—

WHO OWNS THE MEDICAL PAPER? .... 761

EDITORIAL—

THE EFFECT OF FREEDOM UPON THE MENTAL AND PHYSICAL HEALTH OF THE NEGRO OF THE SOUTH .... 763

SELECTIONS AND ABSTRACTS .... 768

MEDICAL ITEMS .... 788

BOOK REVIEWS .... 792

MEDICAL ITEMS—CONTINUED .... x, xii, xiii, xiv, xv

ADVERTISERS' INDEX TO ADVERTISEMENTS .... xvi

CLASSIFIED INDEX TO ADVERTISEMENTS .... xxxi

